# The Direct and Indirect Relationships Within the Extended Trans-contextual Model for Moderate-to-vigorous Physical Activity

**DOI:** 10.3389/fped.2021.666040

**Published:** 2021-04-12

**Authors:** Brigita Mieziene, Arunas Emeljanovas, Vitalija Putriute, Dario Novak

**Affiliations:** ^1^Department of Physical and Social Education, Lithuanian Sports University, Kaunas, Lithuania; ^2^Department of General and Applied Kinesiology, University of Zagreb, Zagreb, Croatia

**Keywords:** high school students, need satisfaction, autonomy support, objective moderate-to-vigorous physical activity, leisure time moderate-to-vigorous physical activity

## Abstract

Given the low levels of physical activity (PA) in adolescence, there are challenges to increasing students' PA outside of the school setting. Thus, researchers emphasize the supportive role that physical education (PE) teachers can play in PA motivation both in and out of school. The aim of the present study was to examine an expanded trans-contextual model (TCM) model for the transit of teachers' perceived support of students' autonomy in terms of contextual and situational motivation in PE to objectively measured moderate-to-vigorous physical activity (MVPA) in PE across different PE contents as well as to the motivational sequence for, and participation in, subjective MVPA during leisure time. This cross-sectional study involved 283 high school students, of whom 43.9% were boys. The autonomous support students received and other motivational factors and objective measures of MVPA in PE and subjective MVPA in leisure time were measured. The results indicate that support for autonomy was significantly and directly related to needs satisfaction (β = 0.61, *p* < 0.001) and indirectly to autonomous motivation in PE (β = 0.19, *p* < 0.001) and leisure time (β = 0.16, *p* < 0.001), intention in PE (β = 0.03, *p* < 0.05) and leisure time (β = 0.07, *p* < 0.001), and leisure time MVPA (β = 0.04, *p* < 0.001), although not MVPA in PE. Gender was a significant covariate for both MVPA in PE (β = −0.62, *p* < 0.001) and MVPA in leisure time (β = −0.37, *p* < 0.001), with higher MVPA in boys than girls. This study filled a gap in the scientific literature by demonstrating the full motivational sequence resulting in actual MVPA in PE classes. It also demonstrated that the main goal of PE of enhancing PA not only in school but also outside of school is working. The main motivator is needs satisfaction based on PE teachers' support.

## Introduction

Physical education (PE) is meant to not only keep children physically active in school but also encourage them to be physically active outside of school (1). Students often fail to engage in a sufficient amount of moderate-to-vigorous PA (MVPA) during PE class (2–4). In general, insufficient physical activity (PA) is a problem among adolescents, as ~80% of adolescents are physically inactive worldwide (5). Only around 30% of high school students meet the World Health Organization's recommendation that they be physically active at a moderate-to-vigorous level for at least 1 h a day (6).

Given the low levels of PA in adolescents, the challenges to increasing their PA outside of school include lack of access to facilities, unsafe neighborhoods, and attractive sedentary alternatives. Hence, maximum active movement time in PE should be a public health priority (3). There is evidence to suggest that students' PA levels vary by PE lesson content (7–9). Researchers also emphasize the supportive role that PE teachers play in PA motivation both in and out of school (10). A systematic review of interventions in PE used to achieve higher MVPA in students suggests that interventions developed in reference to a theory of behavior change, and specifically those that target hypothesized motivational mediators of behavior change, are more successful than interventions that are not grounded in theory (3). Many behavior-change models suggest predictors for health-related behaviors, including PA. Some predictors are present across models, while others are distinct to a particular model. The shortcomings of isolated models indicate the need to integrate them. By integrating models explaining PA, it is possible to not only grasp the factors that explain motivation for PA but also evaluate the sequences (paths) between different predictors (11). This last point is crucial as the main purpose of applying a model or theory is to track how factors relate to behavior rather than simply identify a specific relationship.

### Trans-Contextual Model

The current study aimed to explain how PE motivational factors impact MVPA in PE as well as during high school students' leisure time via the trans-contextual model (TCM) of behavioral change. The primary focus of the TCM is to explain the motivational transition from the PE domain to the leisure time domain triggered by support for autonomy (12). The underlying premise of the TCM is that the predictors of PA are easier to target in the organized context of PE rather than in the leisure time, context; however, benefits can be accrued in both contexts. In addition, the TCM integrates qualitative and quantitative factors from different motivational perspectives.

The TCM integrates two classical behavioral theories—self-determination theory [SDT, (13)] and the theory of planned behavior [TPB, (14)]. The former theory– SDT—analyzes motivation, which is divided into autonomous motivation (self-determined) and controlled motivation (not self-determined). Individuals differ in terms of the manifestations of their autonomous and controlled motivations. However, the degree of autonomy present in different behavioral contexts may vary (e.g., a person can be more autonomous in terms of exercising than in terms of taking medication regularly). The motivational level attained along the self-determination continuum depends upon the levels to which three main psychological needs are satisfied. These needs are autonomy (free will to make self-determined decisions), competence (perception of self-efficacy), and relatedness (close, trusting relationships with significant others), all of which are interdependent (15). In SDT, motivation gained through the satisfaction of psychological needs is affected by the perceived support for autonomy, a certain degree of which can promote or interfere with an individual's self-determination (13). Hence, SDT considers the role of an external agent in an individual's motivational transition. Support for autonomy emphasizes choice, although choices should be provided within specific rules and limits, with rationalizations provided for tasks and limits, acknowledgments of students' feelings and perspectives, respect, opportunities for initiative, and avoidance of controlling behaviors (16). Empirical evidence indicates the positive effect of perceived teacher support for student autonomy on students' motivational outcomes, including outcomes in PE (17–22). It has been determined that perceived teacher support for autonomy is related to higher levels of PA in PE classes ((23)). This transfer is triggered by the motivational process, in which an environment that is supportive of autonomy increases levels of autonomy, competence, relatedness, and self-determination (24). The latter theory—TPB—also includes social-cognitive factors. The central construct of this theory of motivation is intention, which serves an indicator of readiness for action, investment in planned efforts, and future behavior. Intention reflects the amount of motivation an individual has, i.e., how much an individual is motivated to perform a specific behavior. Thus, intention can serve as a generalized quantitative characterization of motivation. Intention largely depends on an individual's attitudes (positive or negative feelings with respect to behavior), subjective norms (the level of motivation arising from the expectations of significant others regarding the individual's involvement in a particular behavior), and perceived behavioral control (beliefs about their self-efficacy with regards to engaging in a behavior) (14).

Some authors have stated that qualitative motivation determines quantitative motivation (12). For instance, in SDT, the qualitative motivation of an individual with an autonomous motivation for physical activity, i.e., their behavioral context, will lead them to be equally motivated to perform various physical activity activities (25). Thus, qualitative motivation is contextual motivation. Meanwhile, TPB identifies the level of readiness or plan for performing a very specific behavior at a certain frequency within certain time limits, for instance, exercising at a particular frequency for a specified period of time. Such motivation is considered to be situational. The integration of the SDT and TPB models, which cover different motivational perspectives, helps in the identification of the correct actors as the scene shifts from context to situation and provides insights for interventions in a targeted, cost-effective, and efficient way. The integration of TPB and SDT to form the TCM is based on following three arguments:

(1) Both TPB and SDT are based on cognitive, i.e., decision-making, processes. Both theories stress that behavior is a conscious decision (26) and not spontaneous.
(2) The theoretical premises of SDT include the preposition that autonomous motivations become intentions regarding behavior via beliefs (27). TPB includes intention as the most proximal predictor of a specific behavior as the result of a set of beliefs but does not explain the more general reasons as to why those beliefs are held (28). The level of self-determination might explain differences in behavioral beliefs (29), as beliefs go along with the level of autonomy (26). However, SDT is not focused on the decisional processes for very specific behavior regarding frequency or time dedicated (29). The integrated model avoids these shortcomings in the two models. In particular, the contextual motivation determined by SDT is related to behavior through the intentional decision-making processes in TPB (26).
(3) TPB is focused on the quantitative aspect of motivation, i.e., the strength of the intention. However, a strong intention does not always turn into actual behavior (30). Hence, reducing motivation to its quantitative dimension is not always justified. Meanwhile, SDT focuses on qualitative differences in motivation. For instance, two students might have the same intentions to be physically active in PE; however, one could be driven by his/her inner motivation, whereas the other student could be driven by pressure from a teacher (31). Thus, including the qualitative aspects of motivation could be helpful in explaining situational behavior in a more rational way.

### The Present Study

Using the TCM to explain the transition of motivation from PE to the leisure domain has received empirical support across multiple studies (25, 32, 33). Classical TCM ignores needs satisfaction, which is the key to enhancing autonomous motivation in SDT. Similar to a study on Spanish adolescents (34), this study broadened TCM to include needs satisfaction. Moreover, few TCM-based studies have examined objectively measured PA (33), specifically objectively measured MVPA in PE. In research similar to this study, the integrated TCM model has explained the transition of motivation from PE to leisure time (25, 33, 35). However, many previous studies have limited their explorations of the transition of motivation for PA in PE to leisure-time motivation and/or actual behavior (33, 36, 37). In particular, very few studies have examined PA in PE alongside leisure-time PA. Finally, none of these studies examined the motivational sequence triggered by teachers' support for autonomy in terms of MVPA measured objectively across PE classes with different educational contents. Meanwhile, researchers have suggested that non-traditional PE class activities might increase students' motivation and engagement in comparison with traditional activities (38). Playing games, practicing skills, and fitness lessons had the greatest impact on MVPA (8). Hence, it is of interest to examine the effect of teachers' support for MVPA on different educational contents in PE classes. **The aim** of the present study was to use the expanded TCM model to explore the transit of perceived teacher support for student autonomy through contextual and situational motivation in PE to objectively measured MVPA in PE across different PE contents as well as to the motivational sequence in the leisure time domain and subjective leisure time MVPA. Thus, the current study attempted to gather information on what has been missed in other studies, namely, the motivational sequence and its transition to PE and leisure-time MVPA. No other study has provided such a full picture to date.

**Figure 1:**
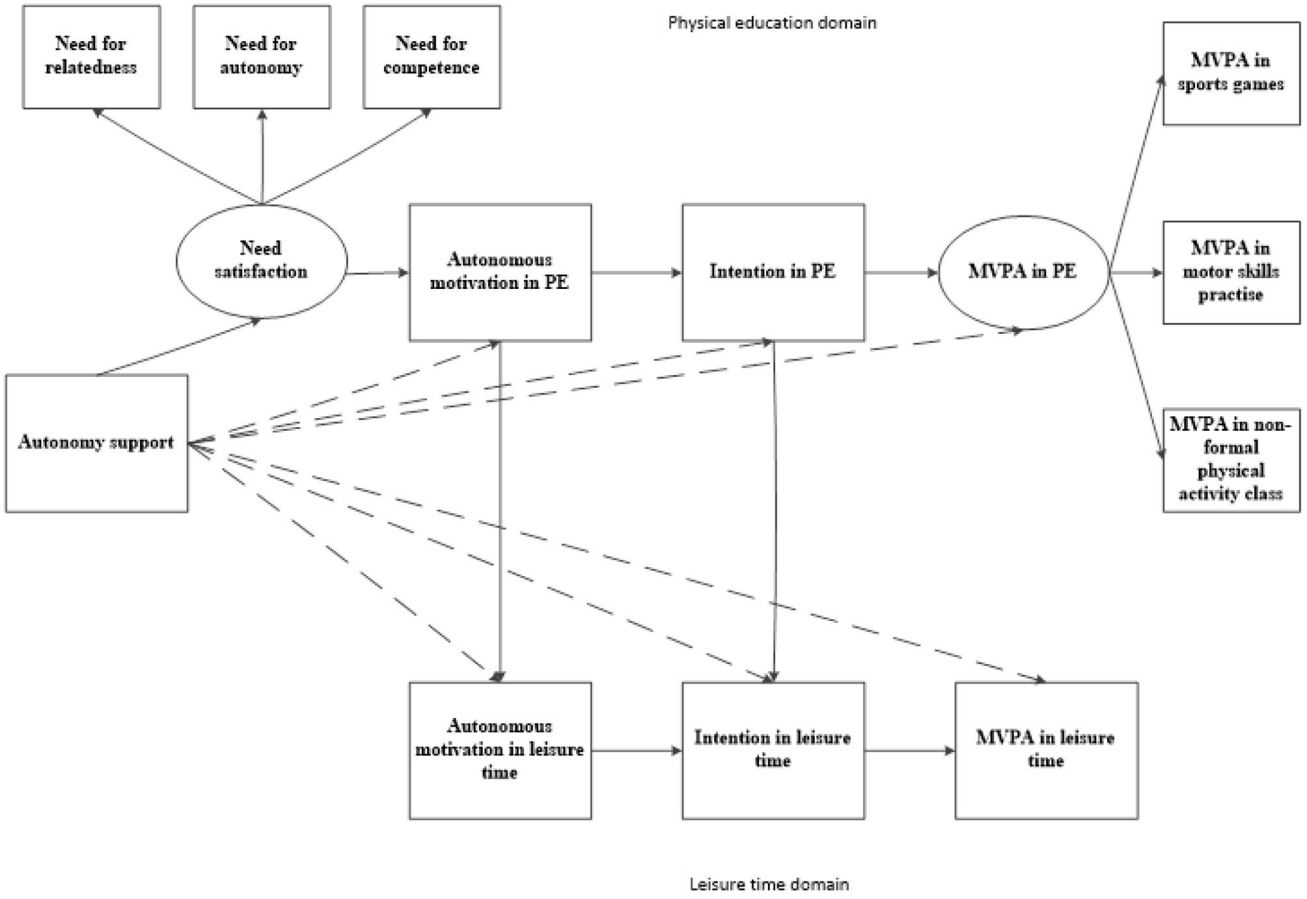
A schematic diagram of the hypothesized relationships in a trans-contextual model. Solid arrows represent the hypothesized direct relationships, whereas the dashed represent the indirect relationships.

Several hypotheses were developed (Figure 1): (1) in the PE domain, PE teacher's perceived support for autonomy will be directly and positively associated with students' needs satisfaction, whereas needs satisfaction will be related to autonomous motivation in PE, autonomous motivation in PE will be associated with the intention to be physically active in PE, and intention in PE will predict MVPA in PE; (2) in the leisure-time domain, autonomous motivation will be related to intention to engage in leisure-time PA, and this intention will be associated with leisure-time MVPA; (3) there are trans-contextual direct relationships between the PE and leisure-time domains, specifically between the contextual autonomous motivations and situational motivations, i.e., intentions, within these two domains; (4) needs satisfaction will be significant mediator in the effect of support for autonomy on MVPA in PE; (5) the indirect effect of perceived support for autonomy will be transferred from PE to the leisure-time domain through other motivational factors arising in PE.

## Methods

### Participants

Initially, 318 students were selected for the study. Due to illness or other reasons, 35 students (11% of the total sample) did not attended one or more PE classes. They were subsequently withdrawn from the study. In the end, this cross-sectional study included 283 high school students. Among them, 43.9 % were boys, and 56.1% were girls. The ages of the participants varied from 15 to 17 years old, with a mean M = 15.62 years, and a standard deviation SD = 0.67 years. Body mass indexes ranged from 14.48 to 29.63, with a mean M = 20.5, and standard deviation SD = 2.54.

### Measures

Support for autonomy in PE was evaluated using a modified version of the short form of the Sport Climate Questionnaire [SCQ, (12)]. The six items on the short form were changed slightly to adapt them to the PE context, for example, “I feel that my PE teacher provides me with choices and options” and “My PE teacher listens to how I would like to do things in PE lessons.” Answers for each item were recorded on a seven-point Likert scale, ranging from 1 (strongly disagree) to 7 (strongly agree). Cronbach's α, which was used to measure the internal consistency of the scale, was 0.808.

Needs satisfaction in PE was assessed in terms of autonomy, competence, and relatedness using 16 items within three respective subscales. The development of the subscales is described in detail elsewhere (21). Satisfaction in terms of autonomy was measured with six scale items (e.g., “I have some choice in what I want to do”), one of which was reverse coded (“I have to force myself to do the activities”). Satisfaction in terms of competence was measured with five scale items (e.g., “I am pretty skilled at PE”). Relatedness was also assessed with five scale items. These items had a common stem, namely, “With the other students in my PE class, I feel….,” which was then followed by terms such as “close,” “valued,” and “supported.” Responses on all 16 items were indicated on a seven-point Likert scale ranging from 1 (“strongly disagree”) to 7 (“strongly agree”) (21). Confirmatory factor analysis (CFA) confirmed the factor structure of the three needs scales, with the exception that the root mean square error of approximation (RMSEA) was above the satisfactory threshold (χ^2^ = 525.99; *df* = 101; RMSE = 0.122; [95% CI= 0.112–0.132]; Comparative fit index (CFI) = 0.971; Tucker–Lewis index (TLI) = 0.965; standardized root mean square residual (SRMR) = 0.057).

Autonomous motivation in PE was identified using a revised version of the Perceived Locus of Causality (PLOC) scale developed by Vlachopoulos et al. (39). This scale was first developed by Ryan and Connell (40), then initially revised by Goudas et al. (41). The scale consists of 19 items. Responses are indicated on a seven-point Likert scale ranging from 1 (“strongly disagree”) to 7 (“strongly agree”). Scores on the Intrinsic, Identified, Introjected, External, and Amotivation scales were summed up individually and divided by the number of items in that scale, then the Relative Autonomy Index in PE was used to calculate the final overall score [(Intrinsic x +2) + (Identified x +1) + (Introjected x−1) + (External x−2) + (Amotivation x−3)]. The CFA demonstrated satisfactory parameters (χ^2^ = 514.47; *df* = 143; RMSE = 0.096 [95% CI = 0.087–0.105]; CFI = 0.978; TLI = 0.974; SRMR = 0.053), confirming the structure of the original scales. The Cronbach α's of the scales ranged from 0.811 to 0.932.

To assess the autonomous motivation for participating in physical activities during leisure time, the BREQ-2 questionnaire was used (42). This 19-item questionnaire is comprised of five subscales that reflect intrinsic, identified, introjected, and external motivation as well as amotivation. Answers are provided on a 5-point Likert scale ranging from 0 = “not true for me” to 4 = “very true for me.” Subscale scores were calculated by summing the individual item scores and dividing by the number of items. For this study, the Relative Autonomy Index (RAI) score, which indicates the degree to which a student's physical activity motivation is autonomous, was used. The RAI is calculated by multiplying each subscale score by a specific ratio, then summing the scores. The RAI score ranges from−24 to +20, with higher positive scores indicating more autonomous motivations (15). CFA was performed and indicated that the parameters were good (χ^2^ = 262.64; *df* = 86; RMSE = 0.042 [95% CI = 0.036–0.048]; CFI = 0.993; TLI = 0.986; SRMR = 0.014), confirming the structure of the original scales. The Cronbach α's of the scales ranged from 0.817 to 0.899.

The intention to participate in PE class and leisure-time PA over a two-week period was assessed with three items designed for leisure-time PA by Chatzisarantis et al. (43) and three similarly-worded items for PE class PA. The items were developed in accordance with the work of Ajzen and Madden (44) and worded to reflect behavioral criteria in terms of time, context, target, and action (45). For instance, “I intend to exercise/play sport at least 3 times a week during the next two weeks” was used for leisure-time PA, and “I intend to exercise/play sport until I sweat at least half a PE class during the upcoming classes” was used for PE class PA. Responses were indicated on a 7-point scale ranging from 1 (very unlikely) to 7 (very likely). The Cronbach α's for leisure-time and PE class PA intention scales were 0.880 and 0.920, respectively.

Physical activity in PE was measured using an accelerometer (Tri-axis ActiTrainer Activity Monitor) validated in other study (46). In accordance with the Lithuanian PE curriculum (47), compulsory PE content should include games (basketball, volleyball, and other sports games as well as general gymnastics), practice in motor skills (correct posture, breathing, and movement when running, carrying an object, pushing, rolling, crawling, and balance, along with intellectual skills, such as concentration, and self-confidence), and non-traditional physical activities (age-appropriate non-traditional, non-Olympic sports: dancing, aerobics, skateboarding, roller skating sports, darts, bowling, weightlifting, yoga, fighting arts, discus, and more). MVPA in these three different areas was identified. The mean percentage of MVPA in these three types of classes for a student was considered to be the indicator of their PA in PE and was calculated as follows. Vertical and horizontal accelerations in motion were counted in epoch time lengths of 60 s. The recorded count for each epoch represented the intensity of the activity undertaken during that time period. Five PA levels were distinguished in accordance with the count obtained during the epochs: sedentary (0-149), light (150-499), moderate (500-3999), vigorous (4000-7599), and very vigorous (7600 and above) (48). Minutes spent in moderate, vigorous, and very vigorous PA were first summed up, then divided by 45 (45-min classes) to obtain percentages.

PA in leisure time was measured using the short form of the International Physical Activity Questionnaire [IPAQ, (49)]. The questionnaire is described in detail elsewhere; however, for the purpose of this study, four items from this 7-item questionnaire were used, specifically those recording the frequency of, and time spent engaged in, vigorous and moderate physical activity (e.g., “During the last 7 days, on how many days did you do moderate physical activities like carrying light loads, bicycling at a regular pace, or doubles tennis? Do not include walking,” and “How much time did you usually spend doing moderate physical activities on one of those days?”). These items showed positive intercorrelations with the objectively measured PA at its respective intensities (50). Weekly minutes spent on vigorous and moderate physical activity were calculated separately by multiplying the number of days per week by the duration on an average day, then summing and dividing by 60 to arrive at hours per week.

Gender was used as the control variable in this study.

### Procedure

The protocol for this study was approved by the Ethics Committee of the Lithuanian Sports University (No. SMTEK-13), and the study was conducted in accordance with the Declaration of Helsinki. Study participants and their parents provided informed consent before participation. The subjective and objective measures were obtained from two schools in the city of Kaunas. Twenty classes in total across two grades (9th and 10th) were used. Students completed the paper questionnaire in the classroom two weeks before the objective measurements were taken. The Researchers explained the aim of, and procedures to be used in, the study prior to the completion of the questionnaire, which took 20–25 min to complete. The objective measurements of MVPA were taken two weeks later than the subjective measurements and across three PE lessons (one game, one motor-skill practice, and one informal physical activity) within the two-week period. Actigraphs were attached to belts by the researchers and worn on students' left hips. All PE classes in Lithuania last 45 min. PA was monitored from the first minute of PE class until class ended (“from bell to bell”). Students changed into their PE clothes prior to the start of each class. However, PE class time included receiving instructions from the PE teachers, during which time the students did not really move; nonetheless, we were still specifically interested in determining the percentage of time students spent in moderate-vigorous activity during their entire “from bell to bell” PE lesson. We followed the same practice for all three PE classes in all schools and for all students.

### Statistical Analysis

Data were analyzed using SPSS 24.0 (SPSS Inc., Chicago, IL, USA) and MPLUS 8.4 software. The means (Ms), standard deviations (SDs), and frequencies of the variables used in the study were measured. Pearson's r was used in correlational analyses. CFA was used to confirm the questionnaire's structure. Structural equation models (SEMs) were used to identify direct and indirect relationships. The χ^2^/df statistic was used as a goodness of fit index for CFA and the SEMs, i.e., the fit was acceptable if 2 < χ^2^/df < 3. In addition, the root mean square error of approximation (RMSEA), along with its 90% confidence interval (CI), was used. The RMSEA is a population-based fit index that is insensitive to the sample size. Moreover, the SRMR, which is a direct assessment of how well and a priori model reproduces the sample data, was employed. Values of RMSE and SRMR < 0.05 were considered to indicate a very good fit, and values < 0.08 were interpreted as indicating a good fit. Also, the comparative fit index (CFI) and Tucker–Lewis index (TLI), both incremental indices used to compare the fit of a hypothesized model with that of a baseline model (i.e., the model with the worst fit), were used. Values of CFI and TLI >0.90 indicate a good model fit, and values >0.95 reflects a very good model fit (51). Statistical significance was set at a *p*-value of less than 0.05.

## Results

Table 1 presents Ms and SDs for the main study variables. The mean value of the support for autonomy variables was just above 4.5 in a range from 1 to 7. The means of the three needs satisfaction variables all approached 5 and had ranges from 1 to 7. The mean for autonomous motivation in PE exceeded 23 points, and the same indicator in leisure time reached nearly nine points. Intentions for MVPA in both PE and leisure had means just above 5. Participation in MVPA in PE classes having different contents averaged about 35% of the 45-min PE class. The mean MVPA in leisure was around 4 h a week.

**Table 1:** Descriptive statistics of study variables.

**Study variables**	**M**	**SD**	**Skewness**	**Kurtosis**
Autonomy support (within 1 to 7)	4.66	1.23	−0.010	−0.434
Need for autonomy (within 1 to 7)	4.79	1.18	−0.129	−0.509
Need for competence (within 1 to 7)	4.83	1.30	−0.308	−0.340
Need for relatedness (within 1 to 7)	5.05	1.60	−0.524	−0.534
RAI in PE	23.31	6.55	0.075	−0.494
RAI in leisure	8.87	6.08	−0.822	0.301
Intention in PE (within 1 to 7)	5.19	1.73	−0.603	−0.694
Intention in leisure (within 1 to 7)	5.38	1.74	−0.777	−0.552
Sports games MVPA (mean %)	36.19	22.34	−0.669	0.083
Motor skills practice MVPA (mean %)	33.85	17.63	−0.366	−0.475
Non-traditional physical activity MVPA (mean %)	34.48	20.85	−0.182	−0.930
Total MVPA in PE (mean %)	35.38	14.75	0.492	−0.404
MVPA in leisure (h/week)	4.04	1.54	−0.490	−0.777

*RAI, Relative Autonomy Index; PE, Physical Education; MVPA, moderate-to-vigorous physical activity; h, hours*.

The correlational analysis presented in Table 2 reveals that support for autonomy was positively related to higher needs satisfaction and especially to a need for relatedness. Support for autonomy was also positively related to autonomous motivation for PA in PE and leisure time, intentions to be physically active in PE and leisure time, and more hours of leisure time MVPA. However, support for autonomy was not related to MVPA in PE, regardless of content. Total MVPA in PE was positively related the need for autonomy, stronger intentions for PA in both PE and leisure, and higher autonomous motivation for leisure-time PA, although these associations were rather weak. It was not positively related to motivation for PA in PE. Meanwhile, hours of MVPA per week were positively associated with all the rest of the variables, with the strongest positive associations showing up for autonomous motivation for leisure time PA and intention to engage in PA during leisure periods.

**Table 2:** Correlations between study variables.

**Study variables**	**1**	**2**	**3**	**4**	**5**	**6**	**7**	**8**	**9**	**10**	**11**	**12**
1.Autonomy support	1											
2. Need for autonomy	0.464^**^	1										
3. Need for competence	0.320^**^	0.521^**^	1									
4. Need for relatedness	0.522^**^	0.508^**^	0.514^**^	1								
5. RAI in PE	0.147^*^	0.512^**^	0.474^**^	0.396^**^	1							
6. RAI in leisure	0.197^**^	0.330^**^	0.471^**^	0.380^**^	0.444^**^	1						
7. Intention in PE	0.178^**^	0.419^**^	0.441^**^	0.348^**^	0.404^**^	0.449^**^	1					
8. Intention in leisure	0.117^*^	0.276^**^	0.397^**^	0.328^**^	0.329^**^	0.642^**^	0.567^**^	1				
9. Sports games MVPA (mean %)	0.073	0.121	0.102	0.025	0.010	0.096	0.025	0.107	1			
10. Motor skills practice MVPA (mean %)	0.009	0.099	0.026	0.068	0.120	0.090	0.032	0.022	0.278^**^	1		
11. Non-formal physical activity MVPA (mean %)	0.044	0.108	0.109	0.107	0.102	0.096	0.021	0.141^*^	0.291^**^	0.339^**^	1	
12. Total MVPA in PE (mean %)	0.017	0.142^*^	0.076	0.006	0.108	0.144^*^	0.138^*^	0.198^**^	0.473^**^	0.528^**^	0.556^**^	1
13. MVPA in leisure (h/week)	0.130^*^	0.167^**^	0.329^**^	0.197^**^	0.238^**^	0.504^**^	0.274^**^	0.568^**^	0.233^**^	0.145^*^	0.206^**^	0.356^**^

*RAI, Relative Autonomy Index; PE, Physical Education; MVPA, moderate-to-vigorous physical activity; h, hours*.

* p<0.05;

** *p<0.01*.

Table 3 presents the direct and mediating effects within the TCM. Two models, one for PE only with three final outcomes represented by MVPA effort by PE class content, and a second one with two final outcomes, namely, latent MVPA in PE and MVPA in leisure time, were developed. The results of the path analysis for the first model involving final outcomes in PE, i.e., MVPA in games, practicing motor skills, and non-traditional physical activities, revealed that the hypothesized model exhibited an acceptable data fit (χ^2^ = 40.00, *df* = 23; CFI = 0.97; TLI = 0.93; RMSEA = 0.055 [90% CI= 0.024–0.083]; SRMR = 0.038). The results of the path analysis for the second model involving final outcomes of latent MVPA in PE and MVPA in leisure time also showed an acceptable fit (χ^2^ = 82.54, *df* = 33; CFI = 0.95; TLI = 0.93; RMSEA = 0.078 [90% CI= 0.057–0.100]; SRMR = 0.049). Latent MVPA in PE was aggregated from the observed MVPAs in classes emphasizing games (β = 0.59), practice in motor skills (β = 0.43), and non-formal physical activities (β = 0.53). The needs satisfaction in each of the models was a latent variable aggregated from the need for autonomy (β = 0.72), competence (β = 0.67), and relatedness (β = 0.75).

**Table 3:** Direct and indirect effects across five models predicting MVPA in sports games, motor skills practice, non-traditional physical activity lessons, total MVPA in physical education and leisure time MVPA.

				**CI (95%)**		
**Independent variable**	**Dependent variable**	**Pathway**	**β**	**LL**	**UL**	***P***
**Direct effects**						
Autonomy support	Latent Need satisfaction	n/a	0.611	0.049	12.722	**<0.001**
Latent Need satisfaction	RAI in PE	n/a	0.311	0.061	5.095	**<0.001**
RAI in PE	Intention in PE	n/a	0.214	0.075	2.843	**0.004**
Intention in PE	Sports games MVPA	n/a	0.137	0.058	2.384	**0.017**
Intention in PE	Motor skills practice MVPA	n/a	0.141	0.061	2.302	**0.021**
Intention in PE	Non-traditional MVPA	n/a	0.053	0.060	0.881	0.378
Intention in PE	Latent MVPA in PE	n/a	0.214	0.075	2.843	**0.004**
RAI in PE	RAI in leisure	n/a	0.813	0.022	36.152	**<0.001**
RAI in leisure	Intention in leisure	n/a	0.471	0.081	5.838	**<0.001**
Intention in PE	Intention in leisure	n/a	0.504	0.046	10.942	**<0.001**
Intention in leisure	MVPA in leisure	n/a	0.533	0.042	12.622	**<0.001**
**Indirect effects**						
Autonomy support	Intention in PE	Latent Need satisfaction, RAI in PE	0.031	0.014	2.270	**0.023**
Autonomy support	Intention in leisure	Latent Need satisfaction, RAI in PE, RAI in leisure	0.073	0.019	3.765	**<0.001**
Autonomy support	RAI in PE	Latent Need satisfaction	0.190	0.041	4.602	**<0.001**
Autonomy support	RAI in leisure	Latent Need satisfaction, RAI in PE	0.155	0.034	4.539	**<0.001**
Autonomy support	Sports games MVPA	Need satisfaction, RAI in PE, Intention in PE	0.007	0.004	1.638	0.101
Autonomy support	Motor skills practice MVPA		0.008	0.005	1.611	0.107
Autonomy support	Non-traditional physical activity MVPA		0.003	0.003	0.821	0.412
Autonomy support	Latent MVPA in PE		0.007	0.004	1.763	0.078
Autonomy support	MVPA in leisure	Need satisfaction, RAI in PE, RAI in leisure, Intention in leisure	0.039	0.011	3.575	**<0.001**
**Covariate effect**						
Gender	Sports games MVPA		−0.803	0.103	−7.796	**<0.001**
Gender	Motor skills practice MVPA		−0.457	−3.827	−3.827	**<0.001**
Gender	Non-traditional MVPA		−0.654	0.112	−5.847	**<0.001**
Gender	Latent MVPA in PE		−0.622	0.068	−9.207	**<0.001**
Gender	MVPA in leisure		−0.368	0.047	−7.871	**<0.001**

*MVPA, moderate-to-vigorous physical activity; PE, Physical Education; RAI, Relative Autonomy Index; n/a, not applicable. Bold p values indicate statistically significant results*.

In the PE domain, direct relationships in the model were observed between support for autonomy and needs satisfaction, needs satisfaction and RAI in PE, RAI and intention in PE, intention in PE and MVPA in games and practicing motor skills but not non-traditional physical activities. Intention in PE also predicted latent MVPA in PE. Meanwhile, RAI in leisure was directly predicted by RAI in PE, revealing a trans-contextual relationship. Further, RAI in leisure time as well as intention in PE predicted intention in leisure, which, in turn, was related to MVPA in leisure, again confirming the trans-contextual effect.

Indirectly, support for autonomy was related to intention in PE, with the path mediated by latent needs satisfaction and RAI in PE, and had a trans-contextual effect on intention in leisure that was mediated by needs satisfaction, RAI in PE, and RAI in leisure. Accordingly, support for autonomy was related to RAI in PE through needs satisfaction and to RAI in leisure through needs satisfaction and RAI in PE. However, support for autonomy was not indirectly related to MVPA in any of the PE classes. It was indirectly associated with MVPA in leisure along a path mediated by needs satisfaction, RAI in PE, RAI in leisure, and intention in leisure. As a covariate, gender was significant for all physical activity indicators, revealing the association of the female gender with lower MVPA in PE, regardless of content, and MVPA in leisure time.

## Discussion

The purpose of the current study was to examine the direct and indirect relationships of motivational factors in the PE and leisure domains and the associations of those motivational factors with objectively measured MVPA in PE class and subjectively measured MVPA in leisure with an expanded TCM. More specifically, a search was made for paths facilitating the transit of the effect of support for autonomy in PE to MVPA in PE across different PE contents and leisure-time PA through contextual and situational motivation in PE and leisure time. Given that PE is the only organized way to encourage PA and that PE teachers are obliged to deliver PE in such a manner as to encourage PA not only in PE classes but in leisure time (1, 52), it is important to track the motivational sequence triggered by teachers that leads to PA. The expanded TCM includes needs satisfaction as a latent variable derived from support for autonomy, competence, and relatedness.

The results showed that students engaged in MVPA for around 35% of the time spent in PE class, varying from 34.48% in non-traditional PA to 36.19% in classes involving games. A recent meta-analysis of studies along this vein indicated very similar results, showing that, on average, students engaged in MVPA during 33.0% of their PE classes (53). Specifically, studies measuring MVPA using accelerometers showed that students spent 34.7% (95% CI = 25.1–44.4%) of a lesson engaging in MVPA (54). Results of other studies suggest that teachers should devote a greater portion of time to games if their aim is to encourage student PA (55).

Gender was a significant covariate in our study, indicating higher MVPA in PE class and leisure time for boys compared to girls. Many studies have also revealed that girls were less physically active in PE classes than boys (56–58). One possible explanation for these results is that male teachers conduct PE lessons with significantly more vigorous activity than female teachers (59). In Lithuanian, girls are taught by female PE instructors, and boys are taught by male PE instructors. The same difference between boys and girls was found in MVPA during leisure time across many countries in a previous cross-national study (60).

### Direct Relationships Within the Physical Education Domain

The current study supports multiple propositions as the result of the use of the expanded TCM. Specifically, higher perceived support for autonomy in PE was directly and positively related to needs satisfaction, which, in turn, was directly and positively related to autonomous motivation in PE. The findings of another recent study revealed that needs for autonomy, competence and relatedness satisfaction were positively related to autonomous motivation (32). Meanwhile, autonomous motivation in PE, which represented contextual motivation in the model, was further related positively to situational motivation, as represented by intention in PE, which is in line with the theoretical presumption that situational motivation is defined by contextual motivation (25). The current study indicated that the intention to participate and be physically active in PE classes predicted objectively measured total MVPA and MVPA in games and practicing motor skills, in particular, but not MVPA in non-traditional physical activity classes. Few studies have dealt with tracking further links to actual PA behavior in PE, especially when it comes to using objective methods (23, 61). Thus, the first premise in this paper mostly confirmed the motivational sequence in PE, except that it failed to validate the intention—MVPA link in the non-traditional PA class relationship. Its failure to do so could be explained by the fact that non-traditional activities are less familiar or not familiar at all to students, meaning that students formed their intentions without knowing what to expect in these PE classes. It is possible that the reality they faced in these classes did not match their intentions. Meanwhile, their intentions to participate in games and practicing motor skills were based on their previous experiences in similar classes, allowing them to formulate more accurate intentions.

### Direct Relationships Within the Leisure-Time Domain

The relationships within the leisure time context were as expected. Autonomous motivation was positively related to intention, and intention was positively related to leisure-time MVPA. Similar results were obtained when attempting to predict objectively measured MVPA in leisure time, although a study of Estonian adolescents found that leisure-time MVPA was not related to MVPA in PE class (62). This finding is in line with results obtained by Ickes et al. (63), who reported that perceived support for autonomy increases intentions to be physically active during free time. Other studies have confirmed the premise that behavior is predicted by intention ((25)). Further, intention can predict leisure-time MVPA, which is in line with the theoretical premise (14) and empirical evidence (64) that intention is the most proximal predictor of behavior. Also, this statement is especially relevant when behavior is self-determined, i.e., based on autonomous motivation (65). Thus, the results of this study confirmed the second hypothesis of this research. Here again, contextual motivation determined situational motivation, which, in turn, led to a specific behavior (25). However, measurements of intention and MVPA in leisure were self-reported, and the relationship between them was remarkably stronger than the relationship between intention and objectively measured MVPA in PE (β = 0.53 vs. β = 0.21, respectively). Hence, the findings regarding the strength of the relationship might have been influenced by the self-reported nature of the measurements. This suggestion is supported by the other studies in which leisure-time MVPA was measured objectively and the strength of the relationship of PA with intention was notably lower (β = 0.11) (33).

### Direct Trans-contextual Links

The direct trans-contextual links within TCM were confirmed, thus validating the third premise of the current study. Namely, autonomous motivation for PA in PE predicted autonomous motivation in leisure time. This finding is in line with Vallerand's (11) theory that autonomous forms of motivation in one context are linked to autonomous motivation for the same kind of behavior in similar contexts and confirmed by empirical evidence (32). These similar but distinct constructs are related when behavior is autonomous in a certain context and subsequently creates motivational representations and anticipated patterns of action in that context that serve as a useful template for motivation and action in closely related contexts (25). This linkage also explains why intention in leisure time can be predicted by intention in PE. These two representations of situational motivation lay within different domains of behavior, with one representing motivation for activities in compulsory PE, and the other representing motivation for out-of-school activities, most likely coming from within. The strong relationship between them again suggests that it is important to pay attention to motivation on a more general level.

### Mediation Effects

The addition of needs satisfaction in the current study as a mediator confirmed the fourth hypothesis of this study, as needs satisfaction, along with autonomous motivation, mediated the indirect relationship between support for autonomy and intention to participate and be physically active in PE classes. In accordance with SDT, teachers' support for students' autonomy in the classroom substantially add to students' needs satisfaction, thus enhancing their autonomous motivations and shifting the locus of causality from the external to the internal (66, 67). However, support for autonomy failed to have an indirect effect on MVPA in PE classes and was not related to MVPA in any of the PE classes when a correlational analysis was performed. These results might reflect the control teachers exert in PE. With a curriculum to follow, teachers cannot avoid using controlling types of behavior in PE, even though they may support autonomy. Another study revealed that despite the support for autonomy, exerting control via rewards in PE has a strong and positive direct link (β = 0.70) to the objectively measured MVPA in PE, while intimidation was negatively related to MVPA in PE (68). These findings indicate that students might confuse rewards in PE with support and were very likely applicable in this study, given the competition involved in games or practicing motor skills. On the other hand, the literature shows that teachers' controlling behaviors are linked to satisfaction with competence (69), which is crucial for furthering the motivational sequence and behavior (70). However, future intervention design studies should explore the direct relationships between support for autonomy and MVPA in PE as well as the motivational sequence facilitating the transit of support for autonomy to actual PA in class.

Moreover, needs satisfaction was an important mediator for the transferring support for autonomy in PE to the leisure-time PA context, namely, autonomous motivation and intention in leisure as well as leisure-time MVPA. While the latent variable needs satisfaction was used in the current study, other studies have found unique mediation effects for the needs for autonomy and competence but not for relatedness satisfaction for autonomous motivation in PE and a mediation effect for the need for competence satisfaction for autonomous motivation in leisure (36). Other studies suggest that perceived support for autonomy is associated with PA in leisure through the motivational sequence, including needs satisfaction and autonomous motivation (71, 72). Even though PA in the PE context has a certain structure, and PA in leisure time is mostly a choice, the shared autonomy in these distinct contexts is partly affected by support for autonomy in PE, which satisfies needs for autonomy (thus providing choice in PE), the competence (thus helping students gain confidence in their abilities to engage in physical activities), and relatedness (thus creating the perception that the one can rely on, trust, and be close to other people.) These perceptions go beyond the PE context, as they are naturally more general. The current study also found an indirect link between support for autonomy in PE and MVPA in leisure time. Hence, the results above are in line with the fifth premise. The transfer of factors leading to increased PA in PE domain to the leisure-time domain can be referred to as nurturing basic psychological needs associated with encouraging PA outside of school (25). Some research has shown that students are more likely to engage in PA outside of school if they perceive pleasure and autonomy in PE lessons (73, 74). Furthermore, the results stress the importance of education, as teachers' encouragement of their students' autonomous motivation during PE is likely to persist in leisure time. Researchers have suggested that in order to improve students' outcomes, it is essential that all students receive positive and satisfying PE experiences (75). In order to identify the magnitude of the effect of support for autonomy t in PE on MVPA in PE, further interventional studies should be performed.

Summing up, the results of this study provide clear indications that needs satisfaction should be included in the TCM, as it is an important predictor of autonomous motivation for PA in PE and leisure time as well as a significant mediator in transmitting the effect of perceived teachers' support for autonomy to other motivational factors in PE and even transferring it to the leisure-time domain. This study also confirmed that psychosocial factors are crucial for behavior. This fact should be considered not only by PE teachers engaged in PE practice but also by PE teacher educators. PE study programs should prepare future teachers to respond to students' needs and enhance their motivation by supporting needs satisfaction.

## Strengths and Limitations

First, among the strengths, objective measurements of MVPA in PE classes were taken. No other study, to the best of our knowledge, has measured PA in PE classes using objective methods when testing TCM. Second, each student's MVPA was measured for three different PE classes, namely, games, practicing motor skills, and non-traditional physical activities, covered the compulsory PE curriculum in Lithuania. Among the limitations, the main one is that leisure-time MVPA was identified via students' subjective evaluations. However, using these measurements should not have compromised the results of the model, given that subjectively measured MVPA correlates with objectively measured MVPA (76). The two-week period between measurements of intention and physical activity was shorter than the four-week period used in other studies. The greater the time period between these measurements, the higher the robustness of the long-range effects under scrutiny (12). However, this fact is more important for intervention studies. Our study studied habitual behavior. Also, as this study was correlational in nature, the causal effects of predictors on outcomes could not be identified. However, in this study, predictors and outcomes were not supposed to change over time. Accordingly, the momentary relationships determined in this study were also supposed to remain the same over time.

## Conclusions

Extending the TCM with needs satisfaction produced two outcomes—objectively measured MVPA in PE and subjectively measured MVPA in leisure time—that met the main theoretical assumptions of the motivational sequence. Namely, perceived PE teacher support for autonomy was directly and positively associated with student needs satisfaction. Needs satisfaction was positively related to autonomous motivation in PE; in turn, the latter was positively associated with intention to be physically active in PE. Intention in PE predicted MVPA in games and motor skills PE classes and the averaged MVPA across three types of PE classes but in non-traditional PE classes. Needs satisfaction mediated the relationship between perceived support for autonomy and autonomous motivation and, alongside with autonomous motivation, the relationship between support for autonomy and intention. The indirect effect of support for autonomy on MVPA in PE was not confirmed. In the leisure-time domain, autonomous motivation time was related to intention to engage in leisure-time PA, and intention was associated with MVPA in leisure time. Trans-contextual relationships between autonomous motivation in PE and the corresponding motivation in leisure time as well as between intention in PE and intention in leisure time were observed. Finally, the indirect effect of support for autonomy on f motivation and MVPA in leisure was captured when mediated through need satisfactions, autonomous motivation, and intention in the PE sequence. The study filled a gap in the scientific literature by demonstrating the full motivational sequence resulting in actual MVPA in PE classes. It also demonstrated that the main goal of PE to enhance PA not only in school but also outside is being fulfilled. The main result is that needs satisfaction must be obtained with PE teachers' support.

## Data Availability Statement

The raw data supporting the conclusions of this article will be made available by the authors, without undue reservation.

## Ethics Statement

The studies involving human participants were reviewed and approved by the Ethics Committee of Lithuanian Sports University (No. SMTEK-13). The study was conducted in accordance with the Declaration of Helsinki. Written informed consent to participate in this study was provided by the participants' legal guardian/next of kin.

## Author Contributions

BM and AE: conception and design. VP and AE: data acquisition. BM and DN: data analysis and interpretation. BM, AE, and VP: drafting the manuscript. DN and AE: critical revision for intellectual content. BM and AE: Administrative, technical or material support. All authors read and approved the final manuscript.

## Conflict of Interest

The authors declare that the research was conducted in the absence of any commercial or financial relationships that could be construed as a potential conflict of interest.
